# Supplementary material for: Marginal ice zone fraction benchmarks sea ice and climate model skill
Source: Nat Commun. 2021 Apr 13;12:2221. doi: 10.1038/s41467-021-22004-7 (PMC8044176; doi:10.1038/s41467-021-22004-7)
Supplement: Supplementary file 1 — Supplementary Information [file 41467_2021_22004_MOESM1_ESM.pdf]

Supplementary Information for: Marginal Ice Zone Fraction Benchmarks Sea Ice and Climate Model Skill

CHRISTOPHER HORVAT

Institute at Brown for Environment and Society, Brown University, Providence, RI, USA

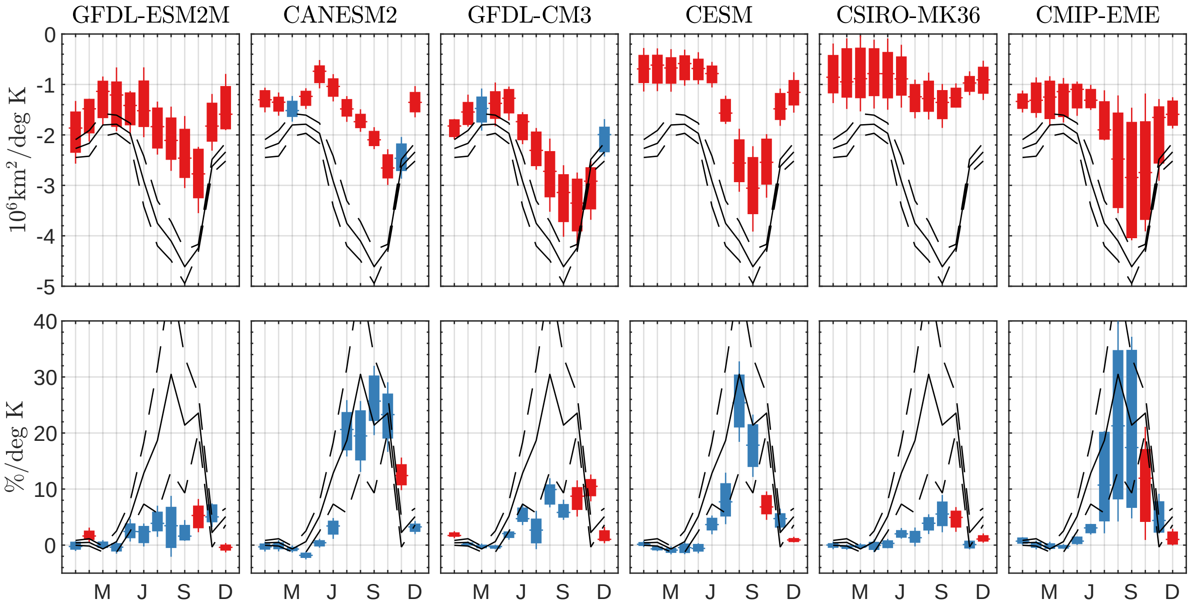

Supplementary Figure 1. Reproduction of Fig. 3 in the main manuscript, but where sea ice sensitivity is computed using the trend in sea ice area loss (units 10^6 km^2/yr) instead of percentage loss of sea ice area per year.

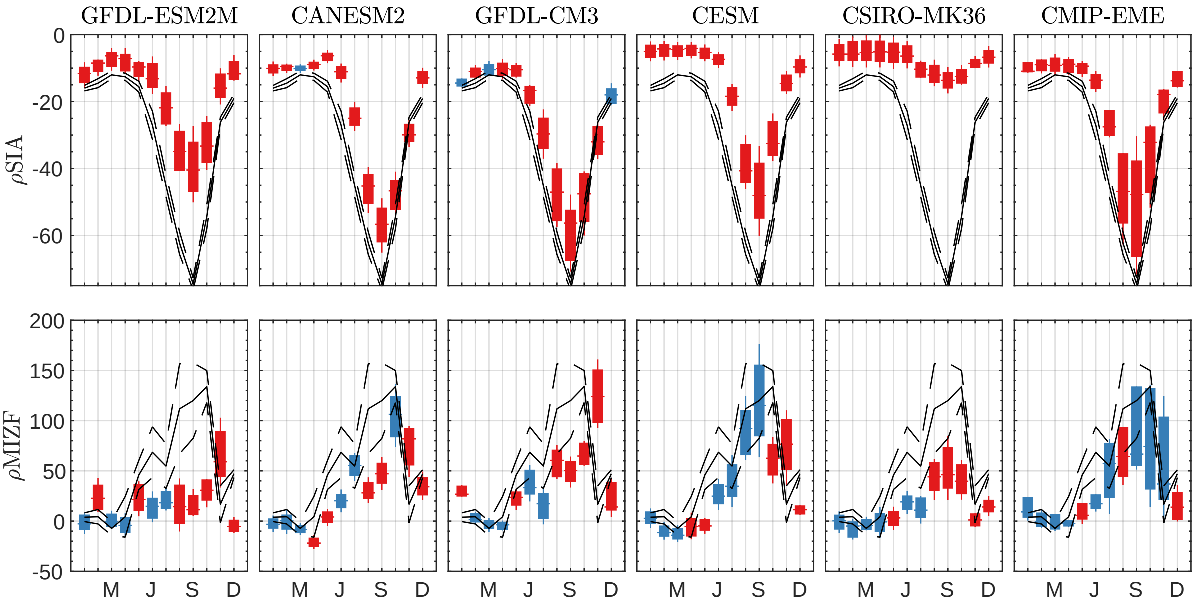

Supplementary Figure 2. Reproduction of Fig. 3 in the main manuscript, but where MIZF is defined to have the same uncertainty characteristics as SIA (see methods).
